# Cost-effectiveness of sofosbuvir in hepatitis C genotype 1 infection in Germany: A reanalysis of published results

**DOI:** 10.1371/journal.pone.0236543

**Published:** 2020-10-02

**Authors:** Afschin Gandjour

**Affiliations:** Frankfurt School of Finance & Management, Frankfurt, Germany; Public Library of Science, UNITED KINGDOM

## Abstract

**Objectives:**

Recently, the results of two economic evaluations were published both of which seemingly demonstrate the cost-effectiveness of sofosbuvir-based regimens for the treatment of chronic hepatitis C genotype 1 infection in Germany. Both analyses were sponsored by the manufacturer of sofosbuvir and use a different methodology: Whereas one evaluation is based on a conventional cost-utility analysis, the other rests upon the efficiency-frontier method used by the German Institute for Quality and Efficiency in Health Care (IQWiG). The purpose of this study is to reanalysis the results of both economic evaluations in combination.

**Design:**

Reanalysis of published decision modelling results.

**Setting:**

Primary care in Germany.

**Participants:**

Patients with chronic hepatitis C genotype 1 infection (treatment-naïve and -experienced, cirrhotic and non-cirrhotic).

**Interventions:**

Sofosbuvir, other anti-hepatitis C virus drugs, and no treatment.

**Primary and secondary outcome measures:**

Cost per unit of health benefit and cost per quality-adjusted life year.

**Results:**

Reanalysis of the results of both economic evaluations in combination reveals an unclear rationale for choosing the selected cost-effectiveness methods as well as a potential publication bias, favoring the product of the manufacturer. Based on the reanalysis, sofosbuvir is not cost-effective in treatment-experienced non-cirrhotic patients, potentially lacks cost-effectiveness in treatment-experienced cirrhotic patients, and is only partially cost-effective in treatment-naïve non-cirrhotic patients. Taken together, these results indicate a lack of cost-effectiveness in three quarters of the German patient population.

**Conclusions:**

Two economic evaluations on sofosbuvir suggest, in combination, that sofosbuvir cannot be considered a cost-effective treatment in three quarters of the German patient population.

## Introduction

In hepatitis C virus (HCV) infections a dual therapy with pegylated interferon and ribavirin had been the standard of care before the first-generation protease inhibitors telaprevir and boceprevir were introduced in Germany in 2011. This was followed by launch of the polymerase inhibitor sofosbuvir in January 2014 and later by second-generation protease inhibitors such as simeprevir.

Sofosbuvir was discovered at Pharmasset, which sold the rights to Gilead Sciences for $11 billion in 2011. Sofosbuvir inhibits the RNA polymerase, which is used by the HCV to replicate its RNA. Compared to previous treatments for HCV infection, sofosbuvir-based regimens overall have a higher cure rate, fewer adverse events, and a reduced duration of therapy. Sofosbuvir is approved in all 6 HCV genotypes. In Germany, the most prevalent HCV genotype is genotype 1, comprising almost 60% of the patient population [1]). Among genotype 1 patients, effectiveness of sofosbuvir varies by subgroup according to the appraisal by the German Federal Joint Committee (Gemeinsamer Bundesausschuss, G-BA) [1], which is responsible for early drug evaluation in Germany: In the largest subgroup, which consists of patients who are treatment-experienced, the German Federal Joint Committee [1] attested no added benefit compared to the less expensive drugs boceprevir and telaprevir. Nevertheless, in treatment-naïve genotype 1 patients the Federal Joint Committee saw a “[h]int of minor additional benefit” compared to boceprevir and telaprevir.

In February 2015, when the price negotiation between Gilead and the National Association of Statutory Health Insurance (SHI) Funds came to an end, both parties agreed on a price of €47,556 for a 12-week treatment net of rebates [2]. In the German SHI system, which covers almost 90% of the German population, the use of sofosbuvir is not restricted to certain genotypes or subgroups such as cirrhotic patients, resulting in approximately 100,000 patients with HCV infection eligible for treatment with sofosbuvir [1]. In 2015, the SHI spent 1.4 billion euros for the treatment of patients with HCV infection. Other regimens for the treatment of HCV infection, which had entered the German market after the launch of sofosbuvir in 2014, were also covered by the expenses [3]. After sales peaked in 2014, annual expenditures for the treatment of HCV infection have decreased due to declining prescription volume [3]. According to the Robert Koch Institute [4], which is responsible for disease control and prevention in Germany, the likely reasons for this decrease are high drug costs and lack of a screening strategy in high-risk target populations such as intravenous drug abusers and prisoners.

The cost of sofosbuvir both at the unit and the population level has raised an intense debate about its cost-effectiveness and affordability in Germany and other countries. Notwithstanding, two recently published model-based cost-effectiveness analyses (CEAs) [2,5,6] both of which were sponsored and commissioned by Gilead and conducted from the perspective of the German health care system report that sofosbuvir is cost-effective in all subgroups of genotype 1 patients. Hence, both studies conclude with a positive view of the price of sofosbuvir. Like any other CEA conducted so far in Germany, they were not used by the German Federal Joint Committee or the National Association of SHI Funds for benefit assessment and pricing. Noteworthy, both CEAs use a different methodology to measure and value health benefits. One CEA [2], which is, strictly speaking, a cost-utility analysis, uses the traditional metric of health outcomes in economic evaluations, the quality-adjusted life year (QALY), and assesses acceptability of incremental cost-per-QALY ratios based on an external willingness-to-pay threshold. In contrast, the other CEA [5,6] uses the results of a discrete-choice experiment, a type of conjoint analysis (CA), to weigh and aggregate the different health outcomes. One fundamental difference between CA and the QALY approach lies in the fact that the latter includes an objective assessment of remaining life expectancy whereas a CA values all attributes including life expectancy subjectively. In addition to relying on the CA method, the second CEA [5,6] employs the so-called efficiency frontier (EF) method to assess cost-effectiveness. The details of this method, which is used by the German Institute for Quality and Efficiency in Health Care (Institut für Qualität und Wirtschaftlichkeit im Gesundheitswesen, IQWiG), are provided in the next section. Both analyses were published in 2017 (January 3 [2], February 16, and June 19 [5,6], respectively). Of note, the two publications by Mühlbacher [5,6] appear to present the same study with only minor differences in the presentation of results. Unfortunately, as the two studies do not cross-reference each other, it is not possible to retrieve information about potential differences from their texts (see “Appraisal of published studies” for a further discussion). We also searched for other studies that had conducted a CEA of different HCV treatment regimens in Germany, used the EF method or QALYs, and, at the same time, were sponsored by Gilead (search in PubMed on February 27, 2020 using the algorithm *hepatitis C AND cost-effectiveness AND Germany*). No other study was found.

The purpose of this paper is to re-analyze the results of the two CEAs and to show that for alternative specifications of the CEA interferon-free sofosbuvir-based regimes (specifically, SOF + LDV ± RBV; see Table 1 for abbreviations) are not cost-effective in three quarters of the German patient population. Note that the reanalysis uses the same prices as the two CEAs. While the price of sofosbuvir in combination with velpatasvir was reduced in April 2018, our analysis is unaffected as this combination regime was not evaluated in the two CEAs. Furthermore, our focus is on the methodology and the conclusions of the two CEAs at the time of their publication.

**Table 1:** Abbreviations of medicines.

BOC	Boceprevir
DAC	Daclatasvir
DSV	Dasabuvir
LDV	Ledipasvir
OMV	Ombitasvir
PegIFN	Peginterferon
PTV/r	Paritaprevir/ritonavir
RBV	Ribavirin
SMV	Simeprevir
SOF	Sofosbuvir
TVR	Telaprevir

## Methods

The sequence of this paper is as follows: First, it presents the EF method according to the specifications by IQWiG; second, it reestimates the EF based on the results presented in Mühlbacher [5,6] by reordering the alternatives and recalculating incremental cost-effectiveness ratios (ICERs); and third, it applies the EF method using the QALY estimates published by Stahmeyer [2].

### Efficiency-frontier method

The Appendix describes the process of appraising new therapeutic entities in Germany and the role of CEA based on the EF method within this process. The EF approach used in this paper follows the requirements by IQWiG [7]. IQWiG’s EF method has been validated both theoretically [8] and empirically (the latter, arguably, in a small-sample study that requires additional confirmation) [9]. To determine reimbursement prices, IQWiG employs the following decision rule (elsewhere also called proportional rule [10,11]): The ICER of a new drug compared to the next effective intervention should not be higher than that of the next effective intervention compared to its next effective intervention. According to IQWiG, the various alternatives are placed on a “cost-benefit plane”, an EF is drawn along non-dominated alternatives, and the reimbursement price is determined by an extension of the last segment of the EF.

According to IQWiG’s methodology, each therapeutic area is assessed separately, i.e., no direct comparisons between therapeutic areas are performed. Although measures of health benefits may differ between therapeutic areas, they need to be the same within a therapeutic area in order to compare the interventions in question. As potential measures of health benefit, IQWiG allows the use of patient-relevant outcomes such as mortality, morbidity (symptoms and complications), and health-related quality of life; validated surrogates of patient-relevant outcomes; and transformations of patient-relevant outcomes into approximately cardinally scaled measures [7]. IQWiG does not explicitly exclude QALYs as a measure of health benefit but criticizes their use on ethical and methodological grounds [7]. Alternatively, IQWiG allows for use of the analytic hierarchy process and CA to weigh the different patient-relevant outcomes. Nevertheless, it also states that as “there are still unsolved methodological problems in the use of these procedures […] it is not planned to use them routinely” [7]. Therefore, both approaches of measuring health benefits, QALYs and CA weights, are viewed with caution.

In terms of costs, IQWiG considers drug-related costs including those for drug acquisition (i.e., pharmacy retail prices net of mandatory rebates for the SHI) and treatment of adverse events; savings from avoided clinical outcomes or events; and change in future healthcare costs due to life prolongation stemming from the reduction in clinical outcomes or events.

### Appraisal of published studies

The CEA by Mühlbacher [5,6] analyzes two subgroups of genotype 1 patients, treatment-naïve and treatment-experienced non-cirrhotic patients. In treatment-experienced non-cirrhotic patients treatment duration of LDV + SOF was 12 weeks whereas in treatment-naïve patients both an 8-week and a 12-week regimen were allowed (which is in the line with the prescribing information).

The EF constructed in Mühlbacher [5,6] deploys medication costs on the cost side and health benefits aggregated by weights obtained from a discrete-choice experiment on the benefit side. Of note are small differences in aggregated benefits between the two publications by Mühlbacher [5,6] that appear only to be the result of different sets of draws in the Monte-Carlo simulation. Unfortunately, the authors do not present base-case outcomes, which are recommended by international guidelines (e.g., [12]; see below) and would be helpful to reconcile the findings. Instead, the authors calculate means across Monte-Carlo simulations. Regrettably, the two published articles do not provide information about this and other potential differences.

The time horizon both of costs and health benefits is limited to the treatment period itself. However, as stated in IQWiG’s method paper [7, p. 79] and partly acknowledged in Mühlbacher [5, p. 271; 6], constructing the EF requires consideration of downstream costs and long-term health benefits. Notwithstanding these omissions, the construction of the EF itself is problematic as it is based on the historical launch sequence of the various treatments (“The EFs are drawn in ascending order according to the chronology of the development stages of the therapies” [5]). That is, the earliest treatment introduced in the market marks the first coordinate of the EF, the next treatment marks the second coordinate, and so forth. Yet, the approach of constructing the EF based on the past launch sequence is not the approach mentioned in IQWiG’s methods paper ([7] as well as prior versions of the methods paper). An EF constructed this way might be appropriate if the goal of the analysis was to analyze, in retrospect, whether prices at the time of the launch were acceptable (i.e., cost-effective). Yet, if the goal of the analysis is to determine the cost-effectiveness at the time of publication, then the EF needs to embrace all available treatments (of note, inflation-adjusted prices at the time of publication are not higher than at launch due to the so-called ‘price moratorium’ in Germany). In fact, [6] confirms the goal of assessing current prices and the need to construct an EF embracing all available treatments (“The new therapies without interferon are *today* [emphasis added] the standard of care [..] but also associated with higher prices. It raises the question to what degree an increased patient benefit justifies higher prices.” (A. Gandjour, Trans.)). Arguably, an analysis of prices at the time of publication is always needed even if the primary goal is to analyze prices at launch. That is, in case historical prices are cost-effective but prices at the time of publication are not, omitting information about the cost-effectiveness of prices at the time of publication can lead to false conclusions by payers and prescribers. As a final remark on this matter, the goal in Mühlbacher [6] was stated to be an assessment of all interferon-free regimens. In contrast, this paper specifically analyzes interferon-free sofosbuvir-based regimes (and in particular SOF + LDV ± RBV) because SOF + LDV is the regimen of commercial interest to the sponsor of the CEA (Gilead). This difference is important to stress as it affects the construction of the EF. That is, when analyzing the cost-effectiveness of interferon-free sofosbuvir-based regimes, these regimes cannot themselves be part of the EF because they need to be evaluated on the basis of the EF (see below for further details).

There is one additional point in the CEA by Mühlbacher [5,6] requiring scrutiny. In treatment-experienced non-cirrhotic patients Mühlbacher [6] do not draw the EF through the coordinates of TVR + PegIFN + RBV over 24 weeks although this regime was part of the historical launch sequence. Its exclusion as a coordinate of the EF is justified by the fact that the regimen is only indicated in patients with a relapse. This subgroup is not small, however, but comprises 38% of all treatment-experienced non-cirrhotic patients, at least as of 2011 [13]. Therefore, the EF plotted in Mühlbacher [6], strictly speaking, only refers to 62% of treatment-experienced non-cirrhotic patients.

Before presenting the results of the reanalysis of the data published by Mühlbacher [5,6], we briefly visit the CEA presented by Stahmeyer et al. [2]. We would like to highlight that it uses QALYs as a measure of health benefit and applies an external willingness-to-pay threshold, thus complying with what is commonly seen as an internationally accepted standard of conducting CEAs. Furthermore, it calculates costs and QALYs over lifetime and uses the EQ-5D-3L questionnaire in order to assess health states by patients. In detail, Stahmeyer et al. [2] analyze four subgroups of genotype 1 patients: 1) treatment-naïve non-cirrhotic patients; 2) treatment-naïve cirrhotic patients; 3) treatment-experienced non-cirrhotic patients; and 4) treatment-experienced cirrhotic patients. In all but one subgroup (treatment-naive non-cirrhotic patients) patients are assumed to receive LDV + SOF over 12 weeks. The authors show cost-effectiveness in all four subgroups.

In order to appraise the quality of reporting of the two economic evaluation studies, we use a 24-item checklist based on the Consolidated Health Economic Evaluation Reporting Standards (CHEERS) statement from the International Society for Pharmacoeconomics and Outcomes Research (ISPOR) Health Economic Evaluation Publication Guidelines Good Reporting Practices Task Force [12]. Following Zakiyah et al. [14], we apply the categories ‘yes’ (Y), ‘no’ (N), ‘partially reported’ (P), and ‘not applicable’ (NA) to assess the quality of reporting, which is based on our interpretation. The ‘partially reported’ category is necessary because some items in the checklist consist of multiple recommendations. As stated by Zakiyah et al. [14], this is subject to bias as the difference between fully reported (Y) and partially reported (P) is not always clear.

### Reanalysis of cost-effectiveness

Given the above criticism on the EF based on a historical launch sequence as published by Mühlbacher [5,6], we draw the EF using all treatments considered in the publication and not just those available at the time of sofosbuvir’s launch. Furthermore, as the analysis needs to assess the cost-effectiveness of interferon-free sofosbuvir-based regimens in particular, it needs to exclude them from the EF itself. We determine ICERs compared to the next effective intervention and rule out interventions that are dominated strictly or by extension. As health benefits in the two publications by Mühlbacher [5,6] vary due to a different set of draws in the Monte-Carlo simulation, we apply the EF method separately to each result.

Moreover, we evaluate the results of Stahmeyer et al. [2] presented in Table 3 of their publication through the lens of IQWiG’s EF method. As the primary goal of our analysis was again to assess the cost-effectiveness of interferon-free sofosbuvir-based regimens (SOF + LDV ± RBV), we do not check the cost-effectiveness of regimens with higher effectiveness. Still, we check whether sofosbuvir-based regimens, if considered cost-effective based on the extrapolation of the last segment of the EF, are dominated by a more effective treatment. As Stahmeyer et al. [2] include ‘no treatment’ as an alternative, which may not be considered an appropriate comparator by the German Federal Joint Committee and may dominate (i.e., rule out) other comparators considered to be appropriate, we analyze in a sensitivity analysis whether excluding ‘no treatment’ as an alternative influences results.

In the final analysis we combine health-benefit data on non-cirrhotic patients by Mühlbacher [6] with the corresponding long-term cost data by Stahmeyer et al. [2] as these are the type of cost data required by IQWiG’s methodology for chronic diseases. In order to appropriately match cost and benefit data in treatment-naive non-cirrhotic patients, a weighted-average health benefit of LDV + SOF over 8 and 12 weeks was calculated (thus matching the long-term cost projection by Stahmeyer et al. for a 8- and 12-week treatment in combination). To this end, we assumed, in agreement with Stahmeyer et al. [2], that 90.7% of treatment-naive patients would qualify for an 8-week treatment. Yet, for some regimens matched data were not available or it was not possible to calculate them (e.g., long-term costs of the treatment combination of BOC + PEG + RBV over 44 weeks plus PEG + RBV over 4 weeks as long-term costs for BOC + PEG + RBV were published for treatment over 48 weeks). These regimens were excluded from the analysis.

## Results

Results of the reanalysis of Mühlbacher [6] are shown in Table 2. In treatment-naïve non-cirrhotic patients the redrawn EF has only two coordinates, PegIFN + RBV and OMV + PTV/r + DSV + RBV. The 8-week treatment with LDV + SOF is cost-effective as it is less effective but not dominated by OMV + PTV/r + DSV + RBV versus PegIFN + RBV. Yet, the 12-week regimen with LDV + SOF is not cost-effective as its ICER is above the extension of the last segment of the EF represented by the ICER of OMV + PTV/r + DSV + RBV versus PegIFN + RBV (€304 per unit of health gain > €14 per unit of health gain). Noteworthy, if the goal of the analysis was not to assess the cost-effectiveness of interferon-free sofosbuvir-based regimens but of the most effective regimen, the EF would be constructed using 8-week treatment with LDV + SOF as a coordinate. In that case, the 12-week regimen with LDV + SOF would still be cost-effective because its ICER compared to OMV + PTV/r + DSV + RBV is lower than the ICER of OMV + PTV/r + DSV + RBV compared to the 8-week treatment with LDV + SOF (€304 per unit of health gain vs. €1041 per unit of health gain). A sensitivity analysis using health benefit data from Mühlbacher [5] confirms the result. The EF again has only two coordinates, PegIFN + RBV and SIM + PegIFN + RBV. The 8-week treatment with LDV + SOF is cost-effective as it dominates by extension SIM + PegIFN + RBV. Yet, the 12-week regimen with LDV + SOF is not cost-effective as its ICER is above the extension of the last segment of the EF represented by the ICER of SIM + PegIFN + RBV versus PegIFN + RBV.

**Table 2:** Reanalysis of the base-case results of the cost-effectiveness analysis by Mühlbacher et al. ([6], Tables 2 and 6). Bold numbers indicate regimes constituting the efficiency frontier (EF). Health benefits represent a “single multidimensional benefit (…) calculated by linear additive aggregation of multiple patient-relevant endpoints” [5].

	Costs (€)	Health benefits	Original EF (ICERs)	Redrawn EF (ICERs)		Costs (€)	Health benefits	Original EF (ICERs)	Redrawn EF (ICERs)
Treatment-naïve, non-cirrhotic					Treatment-experienced, non-cirrhotic				
LDV + SOF 12 weeks	56651	2236.44	7	304	LDV + SOF	56651	2231.15	5	236
OMV + PTV/r + DSV + RBV	50582	2216.47		**14**	OMV + PTV/r + DSV + RBV	50582	2205.45		**14**
LDV + SOF 8 weeks	37768	2204.16	dominant	8	SOF + PegIFN + RBV	52126	1281.11	**7**	strictly dominated
DAC + SOF	72911	1982.70		strictly dominated	TVR + PegIFN + RBV 24 weeks	36333	579.71		dominated by extension
DAC + SOF + RBV	74315	1838.91		strictly dominated	SIM + PegIFN + RBV	46186	454.58		dominated by extension
SOF + PegIFN + RBV	52202	1584.83	**13**	strictly dominated	BOC + PegIFN + RBV, 44 weeks BOC	52353	430.68		strictly dominated
SIM + PegIFN + RBV	36333	1144.99		dominated by extension	TVR + PegIFN + RBV 48 weeks	46186	415.58	**71**	dominated by extension
TVR + PegIFN + RBV	40908	682.95	**32**	strictly dominated	BOC + PegIFN + RBV, 32 weeks BOC	43499	351.52		dominated by extension
BOC + PegIFN + RBV	42661	402.59		strictly dominated	PegIFN + RBV	19706	41.57	**reference**	**reference**
PegIFN + RBV	19706	23.20	**reference**	**reference**					

ICER = incremental cost-effectiveness ratio.

In treatment-experienced non-cirrhotic patients experiencing a relapse, the EF using all available treatments yields that TVR + PegIFN + RBV is dominated by extension by OMV + PTV/r + DSV + RBV (Table 2). That is, both in the subgroup with and without a relapse we obtain the same EF. Based on extrapolation of the last segment of the EF, LDV + SOF is not cost-effective in both subgroups (€236 per unit of health gain > €14 per unit of health gain). Using data on health benefits from Mühlbacher [5], we obtain the same EF and constellation as in Mühlbacher [6], just with different ICERs.

Next, we analyze the results of Stahmeyer et al. [2] presented in Table 3 of their publication through the lens of IQWiG’s EF method. Results are shown in Table 3. In non-cirrhotic patients (Table 3A) results largely confirm those of the reanalysis of the results by Mühlbacher [5,6] shown in Table 2. That is, in treatment-naïve non-cirrhotic patients SOF + LDV is cost-effective when extrapolating the last segment of the EF (€250 per QALY gained < €35,746 per QALY gained). At the same time, SOF + LDV is not dominated by more effective treatment regimens. As a word of caution, as Stahmeyer et al. [2] consider a mix of 8- and 12-week treatment with SOF + LDV, it cannot be excluded that the 12-week treatment, if considered separately, would not be cost-effective (as shown in Table 2 based on data by Mühlbacher [5,6]). In any case, in treatment-experienced non-cirrhotic patients SOF + LDV is again not cost-effective (€27,901 per QALY gained > €24,279 per QALY gained).

**Table 3:** Reanalysis of the base-case results of the cost-effectiveness analysis by Stahmeyer et al. ([2], Table 3).

(a)									
	Costs (€)	QALYs	Original ICERs	Recalculated ICERs		Costs (€)	QALYs	Original ICERs	Recalculated ICERs
Treatment-naïve, non-cirrhotic					Treatment-experienced, non-cirrhotic				
SOF + LDV	41056	20.031	dominant	250	SOF + LDV	57937	18.676	26426	27901
SOF + SMV	77398	19.971	75541	strictly dominated	SOF + SMV	77165	18.626	67563	strictly dominated
SOF + PegIFN + RBV	53999	19.891	29151	strictly dominated	SOF + PegIFN + RBV	53956	18.296	62251	strictly dominated
SMV + PegIFN + RBV	41322	19.629	dominant	strictly dominated	SMV + PegIFN + RBV	44433	18.192	15552	24279
TVR + PegIFN + RBV	43073	19.516	reference	strictly dominated	TVR + PegIFN + RBV	43417	18.127	reference	dominated by extension
SOF + RBV	103696	19.307	strictly dominated	strictly dominated	BOC + PegIFN + RBV	43754	17.991	strictly dominated	strictly dominated
BOC + PegIFN + RBV	40853	19.220	7482	35746	PegIFN + RBV	19314	17.098	23436	dominated by extension
PegIFN + RBV	23981	18.748	24867	13546	No treatment	10515	16.795	24701	reference
No treatment	11559	17.831	18704	reference					
(b)									
	Costs (€)	QALYs	Original ICERs	Recalculated ICERs		Costs (€)	QALYs	Original ICERs	Recalculated ICERs
Treatment-naïve, cirrhotic					Treatment-experienced, cirrhotic				
SOF + LDV + RBV	93185	14.429	3383	2443	SOF + LDV + RBV	91423	13.637	1397	5425
PTV/r/OMV/DSV + RBV	111178	14.422	9428	strictly dominated	SOF + SMV	108985	13.448	7934	strictly dominated
SOF + SMV	111136	14.092	10589	strictly dominated	SOF + DCV	109231	13.041	9430	strictly dominated
SOF + PegIFN + RBV	90376	13.279	3972	7185	SOF + PegIFN + RBV	90201	12.044	2155	dominated by extension
SMV + PegIFN + RBV	80425	11.894	dominant	6181	SMV + PegIFN + RBV	82075	11.914	dominant	6682
BOC + PegIFN + RBV	85478	11.638	12256	strictly dominated	TVR + PegIFN + RBV	87349	10.721	reference	strictly dominated
TVR + PegIFN + RBV	83080	11.442	reference	strictly dominated	BOC + PegIFN + RBV	88664	10.015	strictly dominated	strictly dominated
SOF + RBV	146371	10.268	strictly dominated	strictly dominated	PegIFN + RBV	64254	8.376	9849	dominated by extension
PegIFN + RBV	66446	9.516	8635	dominated by extension	No treatment	53410	7.624	10961	reference
No treatment	54737	7.738	7651	reference					

QALY = quality-adjusted life year.

ICER = incremental cost-effectiveness ratio.

In cirrhotic patients (Table 3B) SOF + LDV is cost-effective based on extrapolation of the EF in both treatment-experienced and non-experienced patients (and is not dominated by more effective regimens). However, excluding ‘no treatment’ as an alternative and using PegIFN + RBV’s coordinates as the origin of the EF leads to a lack of cost-effectiveness of SOF + LDV in treatment-experienced cirrhotic patients. For the other 3 subgroups no change in cost-effectiveness results upon exclusion of ‘no treatment’.

When combining health-benefit data on non-cirrhotic patients by Mühlbacher [6] with the corresponding long-term cost data by Stahmeyer et al. [2], we obtain the results shown in Table 4. They confirm those of the above analyses: In treatment-naïve non-cirrhotic patients LDV + SOF is cost-effective whereas in treatment-experienced non-cirrhotic patients it is not.

**Table 4:** Reanalysis of the base-case results of the cost-effectiveness analysis by Mühlbacher et al. [5,6] using long-term cost data by Stahmeyer et al. [2]. Health benefits represent a “single multidimensional benefit (…) calculated by linear additive aggregation of multiple patient-relevant endpoints” [5].

	Costs (€)	Health benefits	ICER		Costs (€)	Health benefits	ICER
Treatment-naïve, non-cirrhotic				Treatment-experienced, non-cirrhotic			
LDV + SOF	41056	2207.16	0.1	LDV + SOF	57937	2231.15	224
SOF + PegIFN + RBV	53999	1584.83	strictly dominated	OMV + PTV/r + DSV + RBV	52172	2205.45	15
SMV + PegIFN + RBV	41322	1144.99	strictly dominated	SOF + PegIFN + RBV	53956	1281.11	strictly dominated
TVR + PegIFN + RBV	43073	682.95	strictly dominated	SMV + PegIFN + RBV	44433	454.58	dominated by extension
BOC + PegIFN + RBV	40853	402.59	44	PegIFN + RBV	19314	41.57	reference
PegIFN + RBV	23981	23.20	reference				

ICER = incremental cost-effectiveness ratio.

Finally, results of the appraisal of the two economic evaluations are reported in Table 5. As is shown, the study by Stahmeyer et al. [2] largely complies with the CHEERS checklist. Still, it does not provide information on the methods used for identifying the underlying clinical trials as well as their study designs, as requested by item 11 of the CHEERS checklist. This weakness is shared by Mühlbacher [6].

**Table 5:** Appraisal of economic evaluations based on the Consolidated Health Economic Evaluation Reporting Standards (CHEERS) checklist.

	Mühlbacher [5]	Stahmeyer [2]
1. Identified as an economic evaluation	P	Y
2. Structured abstract	Y	Y
3. Clearly stated context	P	P
4. Target population described	P	P
5. Setting and location of decision described	P	P
6. Perspective stated	N	P
7. Comparators described	Y	Y
8. Horizon described	P	Y
9. Discount rate stated for benefits and costs	N	Y
10. Health outcomes described	Y	P
11. Source of effectiveness data described	N	N
12. Measurement and valuation of preference-based outcomes described	N	P
13. Resources and costs described	P	P
14. Currency, price, and conversions reported	NA	Y
15. Model described	NA	P
16. Assumptions described	NA	P
17. Analytical methods described	Y	Y
18. Study parameters described	N	Y
19. Incremental costs and outcomes reported	N	Y
20. Uncertainty characterized	Y	Y
21. Differences between subgroups described	Y	Y
22. Findings, limitations, generalizability, and current knowledge described	P	P
23. Sources of funding stated	Y	Y
24. Conflicts of interest stated	Y	Y

Y = yes, N = no, P = partially reported, NA = not applicable.

## Discussion

Results of the reanalysis of two recently published German CEAs on HCV genotype 1 patients [2,5,6] can be summarized as follows: Unlike the conclusions of the two CEAs, sofosbuvir-based regimens cannot be considered cost-effective in treatment-experienced non-cirrhotic genotype 1 patients and potentially lack cost-effectiveness in treatment-experienced cirrhotic patients. In treatment-naïve non-cirrhotic patients, the 12-week regimen with LDV + SOF is not cost-effective either.

Genotype 1 represents the most prevalent HCV genotype in Germany, comprising an estimated 58% of the patient population with HCV infection [1]. Among genotype 1 patients the largest subgroup is formed by treatment-experienced patients, comprising approximately 74% of the population [1]. Thereof, 96% is estimated to be non-cirrhotic [15], amounting to 71% of the total HCV genotype 1 population. The subgroup of treatment-naïve non-cirrhotic patients (in 9% of which the 12-week regimen is not cost-effective) essentially comprises the remaining population [1]. Therefore, it can be concluded that for three quarters of the analyzed patient population (71% + 2%), sofosbuvir-based regimens cannot be considered cost-effective based on the published data. This means that in these patients the price of sofosbuvir is too high to provide acceptable value. It is a matter of speculation whether the price of sofosbuvir may still be acceptable if interpreted as a weighted-average across all subgroups. As stated in IQWiG’s methods paper [7, p. 83], such calculation is left to the discretion of the decision maker. In any case, as was shown by Stahmeyer [2] already, sofosbuvir’s price is too high for the drug to pay for itself, despite the savings from avoiding liver-related diseases, e.g., liver cirrhosis and hepatocellular carcinoma, as well as liver transplants (while Stahmeyer [2] claim savings in treatment-naïve non-cirrhotic patients, they do not consider all available comparators). Under budgetary restrictions that also exist in Germany sofosbuvir thus leads to opportunity costs and forgone health benefits in other areas of the German health care system.

While IQWiG [7] has treated both QALYs and CA with caution, it clearly supports the use of long-term data on costs and health benefits in the analysis of chronic diseases. Therefore, application of the EF method using the lifetime data reported in Stahmeyer [2] is more in line with the requirements by IQWiG than the use of the short-term data by Mühlbacher [5,6]. Still, in the study by Stahmeyer [2] weighting of the dimensions of the EQ-5D questionnaire by preferences of the general population is likely to be challenged by IQWiG as weights may not be representative for the HCV patient population.

Absent of agreed standards for economic evaluation, the use of QALYs in a traditional CEA framework (which is not accepted by IQWiG) yields a favorable cost-effectiveness ratio [2] (leaving issues around the lack of agreement on a willingness-to-pay threshold in Germany aside [7, p. 102]). But as shown in the EF framework, the very same cost and outcome outputs from traditional CEA are not able to confirm cost-effectiveness in the majority of patients. Clearly, the omission of a QALY-based EF approach raises the issue of a publication or reporting bias with failure to report undesirable results [16].

A few words of caution remain. First, as shown by the appraisal of the quality of the economic evaluations, both economic evaluations use effectiveness data from clinical trials but do not provide information on the methods used for identifying the included studies as well as their study designs, as requested by item 11 of the CHEERS checklist. Hence, we cannot comment on whether more appropriate model clinical input data would shift the EF and hence lead to a different assessment of prices. A similar reservation holds with regard to the discrete-choice experiment underlying the CEA by Mühlbacher [5,6], which is based on an additive utility model. That is, a potentially more appropriate model may cause a shift of the EF. On a related point, both CEAs [2,5,6] did not take the benefit appraisal by the Federal Joint Committee [1] into consideration and did not even discuss or mention it (as stated in the introduction, treatment-experienced genotype 1 patients were not attested an added benefit). The more skeptical viewpoint by the Federal Joint Committee is also shared by a recent review of the Cochrane Collaboration [17], which was not yet available at the time of publication of both CEAs though. Considering the health benefit appraisal by the Federal Joint Committee would result in an even less favorable assessment of the price of sofosbuvir based on the EF method (cf. [18]) and would cast further doubts on the cost-effectiveness of sofosbuvir in treatment-experienced patients. Second, some of the comparators considered in the CEAs may not be considered appropriate according to the criteria of the Federal Joint Committee (e.g., PegIFN + RBV and no treatment) and therefore may need to be excluded from the EF. Third, we present results only for the base case and considering the uncertainty in input parameters may change the shape of the EF [7, p. 109]. This may modify the result and yield, e.g., some probability that treating experienced non-cirrhotic patients is cost-effective and some probability that treating naïve cirrhotic patients is not cost-effective. Drawing the EFs under uncertainty requires access to the original decision models, however. Nevertheless, for the purpose of demonstrating how results can flip when changing the analytical approach, the base-case analysis presented in this paper should suffice. As a side note, the uncertainty around changes in downstream costs as a consequence of changes in sustained virological response (SVR) cancels out when applying the EF method because the relationship between change in SVR and change in downstream costs is the same for all comparators (i.e., the ratio is the same regardless of how effective the intervention is, cf. Gandjour [19]). Fourth, it is possible to separately draw an EF for each endpoint such as SVR and then “weigh” endpoint-specific prices without reverting to QALYs or CA [7, p. 83]. Using this approach could yield different results than the QALY- or CA-based approach deployed in the published CEAs and this paper. And fifth, changes in drug prices after publication of the two economic evaluations were not considered for the reasons mentioned in the introduction.

Based on the findings revealed in this paper it is recommended that IQWiG—through its future methodological updates—explicitly states how to draw and extrapolate the EF when the goal is not to assess the price of the most effective treatment. Until analytical controversies are resolved, it might be useful to require prospective registration of CEAs in order to avoid reporting and publication biases (cf. [16]).

## Appendix

In Germany, new legislation regulating the reimbursement of new, innovative medicines within the statutory healthcare system (Arzneimittelmarktneuordnungsgesetz) was introduced on 1 January 2011 [20]. According to this law, new products are subject to an early benefit assessment to determine whether there is sufficient evidence of added medical benefits overall and in particular patient subgroups compared to appropriate therapeutic alternatives. If such added benefits are confirmed, manufacturers and representatives of the statutory health insurance (SHI) are expected to agree on an appropriate reimbursement price within 6 months, starting from the completion of the benefit appraisal by the German Federal Joint Committee. If drug makers and health insurers cannot agree on the price, a final decision on the reimbursement price will be made by an arbitration body. If one of the parties involved wishes so, the Institute for Quality and Efficiency in Health Care (IQWiG) will be commissioned with a formal evaluation of costs and benefits of the product in question.
